# Body Mass Index Is Associated with Inflammatory Bowel Disease: A Systematic Review and Meta-Analysis

**DOI:** 10.1371/journal.pone.0144872

**Published:** 2015-12-14

**Authors:** Jie Dong, Yi Chen, Yuchen Tang, Fei Xu, Chaohui Yu, Youming Li, Prasoon Pankaj, Ning Dai

**Affiliations:** 1 Department of Gastroenterology, Sir Run Run Shaw Hospital, Zhejiang University School of Medicine, Hangzhou, Zhejiang, China; 2 Department of Gastroenterology, the First Affiliated Hospital, Zhejiang University School of Medicine, Hangzhou, Zhejiang, China; 3 Departments of Endocrinology, Sir Run Run Shaw Hospital, Zhejiang University School of Medicine, Hangzhou, Zhejiang, China; 4 Department of Hepatobiliary and Pancreatic Surgery, the Second Affiliated Hospital, Zhejiang University School of Medicine, Hangzhou, Zhejiang, China; University Hospital Llandough, UNITED KINGDOM

## Abstract

**Background:**

Prior work suggested that patients with inflammatory bowel diseases (IBD) have lower body mass index (BMI) than controls and patients with lower BMI have more serious complications.

**Goal:**

The study was aimed to find relationship between BMI in patients with and without IBD, investigate effects of medicine therapy and disease stages on patients’ BMI.

**Methods:**

Potentially eligible studies were identified through searching PubMed, Cochrane and Embase databases. Outcome measurements of mean BMI and the number of patients from each study were pooled by a random-effect model. Publication bias test, sensitivity analysis and subgroup analysis were conducted.

**Results:**

A total of 24 studies containing 1442 patients and 2059 controls were included. Main results were as follows: (1) BMI in Crohn’s disease (CD) patients was lower than that in health controls (-1.88, 95% CI -2.77 to -1.00, *P*< 0.001); (2) Medical therapy significantly improved BMI of CD patients (with therapy: -1.58, -3.33 to 0.16; without: -2.09, 95% CI -3.21 to -0.98) while on the contrary not significantly improving BMI of UC patients (with therapy: -0.24, 95% CI -3.68 to 3.20; without: -1.34, 95% CI -2.87 to 0.20, *P* = 0.57); (3) Both CD and UC patients in active phase showed significantly greater BMI difference compared with controls than those in remission (CD patients: remission: -2.25, 95% CI -3.38 to -1.11; active phase: -4.25, 95% CI -5.58 to -2.92, *P* = 0.03; UC patients: remission: 0.4, 95% CI -2.05 to 2.84; active phase: -5.38, -6.78 to -3.97, *P* = 0.001).

**Conclusions:**

BMI is lower in CD patients; medical therapy couldn’t improve BMI of IBD patients; the state of disease affects BMI of CD patients and UC patients.

## Introduction

Inflammatory bowel diseases (IBD), consisting of crohn’s disease (CD) and ulcerative colitis (UC), is chronic inflammatory diseases with the gastrointestinal tract [1]. Pathophysiology of IBD is yet not fully apprehended, and it has been found related to overactive mucosal immune system within the bowel [2]. During recent decades, incidence of IBD in traditionally high incidence areas, such as the United States and Europe, has been relatively stable. While the incidence of IBD has been increasing in previously low incidence areas, including China [3], it highly advocates that a tremendous epidemic of IBD is coming and more contemplation should be paid to IBD prevention, diagnosis, treatment as well as prognosis.

Recently, another great health threat developed. Global epidemic of overweight and obesity—"globesity"—is rapidly becoming major public health problem and obesity has been leading to excess morbidity and mortality. Body mass index (BMI) is a simple index of weight-for-height commonly used to classify underweight, overweight and obesity in adults. BMI has been found related with numerous health conditions, such as cancers [4–9], osteoarthritis [10], obstructive sleep apnea[11], non-alcoholic fatty liver disease [12], gallbladder diseases [13], et al.

The relationship between body mass index (BMI) and factors of inflammatory bowel diseases (IBD), such as morbidity, complications, prognosis, the stage of the disease, medical therapy, has been reported in many studies. Some studies have discovered that in IBD patients, BMI were lower than that in non-IBD controls [14–17]. However, others indicated that UC patients have higher BMI than controls or have BMI in the normal range [18, 19]. Some studies [15, 17, 19–23] show that medical therapy could decrease lean mass, while others indicated that patients’ BMI is lower than controls’ before the use of therapy [24]. Decreased BMI was found in patients with active UC, but was not commonly observed in CD patients compared with healthy controls. Nevertheless, some studies pointed out that patients with inactive CD having a lower BMI than both UC and healthy controls [25]. Since a low BMI is associated with negative health outcomes and conclusions about BMI in IBD patients were inconsistent, we did a systematic review of all relevant literature, and pooled analysis of BMI in IBD patients versus controls in order to find out that: 1) whether there is a relationship between BMI and IBD, 2) whether medical therapies and the stage of disease have effect on patients’ BMI.

## Materials and Methods

### Data sources, search strategy, and selection criteria

Two authors (Jie Dong and Yi Chen) independently searched PubMed, Cochrane and Embase databases (up to October 2015) for eligible studies. The core search consisted of terms related to body mass index (“overweight”, “body weight”, “obesity”, “BMI”, “body mass index”, “body surface area”, “body size”, “adiposity”, and “fat”). These terms were combined with terms of IBD (“IBD”, “inflammatory bowel disease”, “crohn”, “ulcerative colitis”).

All articles and relevant reviews included in this study were screened [26, 27] for potential missing studies. After eliminating duplicate studies, titles and abstracts of all articles obtained were screened by Jie Dong and Yi Chen to exclude the irrelevant. The remaining articles were read thoroughly and those who met selection criteria were included. Divergence were resolved by consulting with the third author, Ning Dai.

Inclusion criteria were as follows: (1) studies published as epidemiology studies or clinical trials evaluating association between BMI and IBD, concerning risk of IBD in different BMI patients, effect of BMI on diagnosis or prognosis in IBD patients or merely reported BMI in both IBD patients and health controls; (2) BMI of patients were provided or could be calculated in form of Mean±SD; (3) BMI of controls and BMI of IBD patients were not matched.

#### Data extraction and quality assessment

The following information was extracted: the first author, year of publication, study aim, gender, medicine usage, assessment of disease activity, source of BMI and definition of controls, number, age and BMI of both patients and controls. Extracted data were looked over to consolidate by two authors Jie Dong and Yi Chen. Discrepancies were resolved by the third investigator, Ning Dai.

If a study did not incorporate enough data to be included in the meta-analysis (i.e., no risk estimates and/or 95% confidence intervals or no specific BMI in patients or controls), the corresponding author would be contacted via email and the missing data would be solicited at least twice.

The quality of each study was assessed according to Newcastle-Ottawa quality assessment scale [28]. This scale consists of three factors: patient selection, comparability of study groups, and assessment of outcomes. A score of 0–9 (labeled as stars) was used to indicate the quality of each study.

### Data Synthesis and Statistic Analysis

All analyses were conducted using the STATA software (version 12, STATA Corporation, College Station, TX, U.S.). Publication bias was conducted through Begg’s adjusted rank correlation test.[29] Random effect meta-analysis was employed to compare difference in BMI between IBD patients and healthy controls in the form of weighted mean difference (WMD). Statistical heterogeneity among studies was evaluated though Cochran’s Q test and I^2^ statistic [30]. The extent of heterogeneity across studies was checked using the chi-square test and I^2^ test; *P*≤0.10 and/or I^2^>50% indicates significant heterogeneity. If so, subgroup analysis will be conducted. Sensitivity analysis was performed to investigate the contribution of each study to the heterogeneity by comparing results before and after sequentially removing one study and reanalyzing the pooled estimate for remaining studies.

## Results

### Search results and Study characteristics

**Fig 1:**
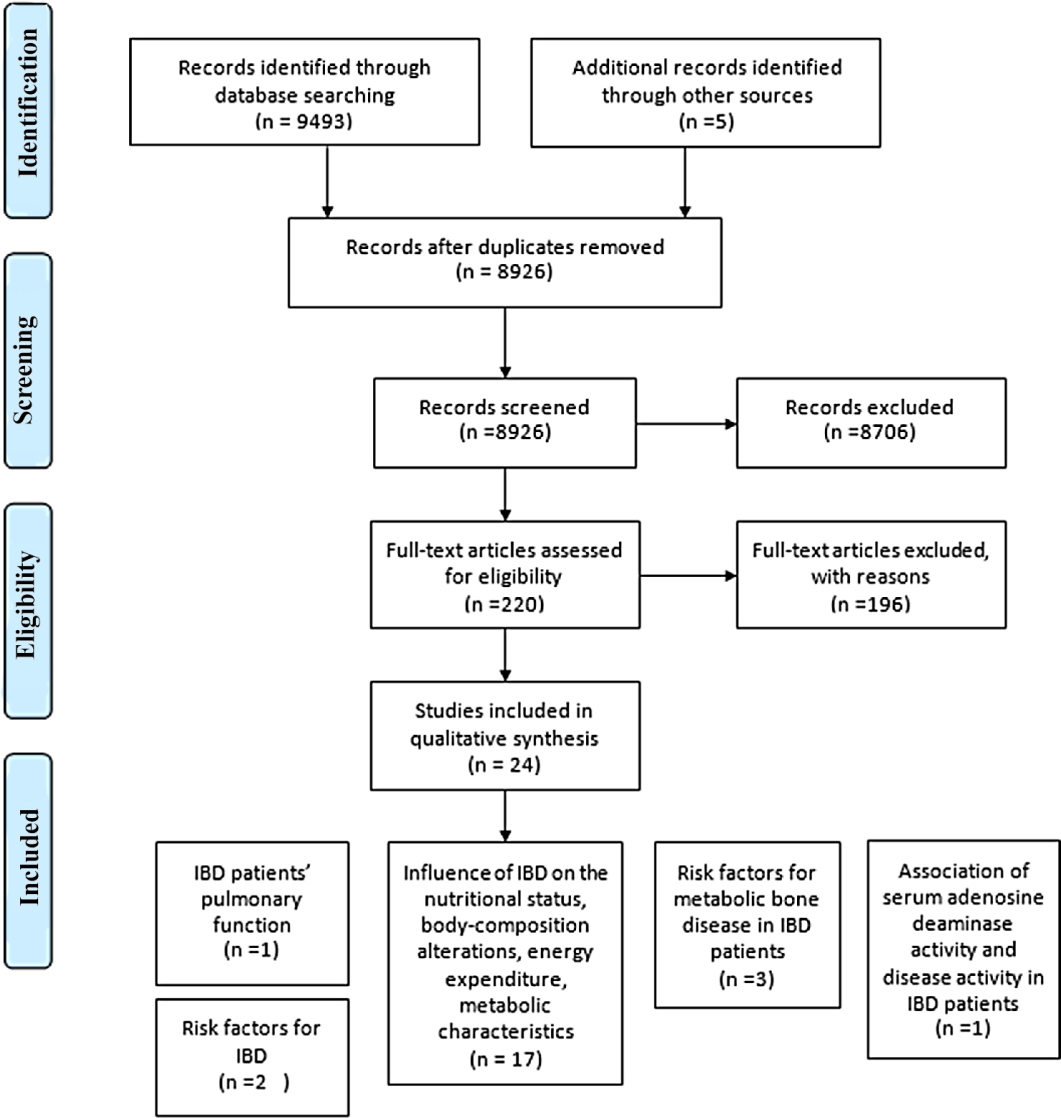
Flow diagram of systematic literature search on BMI and IBD.

9,493 potential relevant articles were identified by searching the databases. An additional five studies were included from reference retrieve. After eliminating duplicates, 8,926 papers were screened. With reading titles and abstracts, a total of 220 articles were considered potentially eligible and full texts were carefully reviewed for inclusion. Among these articles, 196 were subsequently excluded for the following reasons: 173 did not investigate the relationship between BMI and IBD; 13 did not provide sufficient data and did not reply to us; in five studies, BMI couldn’t be calculated in form of Mean±SD; in another five articles, BMI of controls were matched with patients’ BMI. Thus, a total of 24 articles were finally included (Fig 1). 19 articles focus on Europeans, one on Americans, two on Asians, one on Australians and one on Africans. Study designs included three prospective cohort studies, four cross-sectional studies and 17 case–control studies. 10 assessed BMI in UC patients [15, 16, 18, 19, 21, 25, 31–34], 21 enrolled CD patients [15, 17–25, 31–33, 35–43] and three included patients of the undefined IBD type [14, 15, 33]. A total of seven studies investigated the effect of medical therapy on BMI improvement [15, 17, 19–23]. For IBD state, four studies assessed patients in active phase [15, 16, 37, 43], while 12 evaluated patients in clinical remission and 9 reported both [16, 17, 23, 25, 31, 35, 36, 38, 40–43].

The aims and quality assessments of these studies and baseline characteristics of patients and controls are specified in Table 1.

**Table 1:** Aim and definition of the study population of studies that reported the Body Mass Index (BMI) of patients with inflammatory bowel disease (IBD) and healthy controls.

First author, year	Aim	Patients definition	Controls definition	Medicine usage	Assessment of disease activity	Source of BMI	Patients	Controls	Patients age^a^ (years)	Controls age^a^ (years)	BMI^a^ / patients	BMI^a^ / controls	NOS	Study design	Participants’ region
1. Chan, S. S. M. 2013	Perform the first prospective cohort study investigating if there is an association between obesity and the development of incident IBD.	Men and women aged 20–80 years with CD or UC	Four randomly selected controls for age at recruitment, gender, and date of recruitment into the study	Not mentioned	Not mentioned	BMI was calculated from weight and height	CD:75 UC:177	CD:300UC:708	CD: 50.0 (24.0–69.2) UC: 52.8 (22.0–77.0) ^c^	CD: 49.8 (24.0–69.0) UC: 52.7 (22.0–77.2) ^c^	CD: 25.1±3.8 UC: 25.4±3.7	CD: 25.1±3.9 UC: 25.8±4.0	8	Prospective Cohort Study	European
2. Burnham, J. M. 2005	Quantify lean and fat mass in children and young adults with CD and in healthy control subjects, relative to height and pubertal maturation.	Persons aged 4–25 y with CD, PCDAI at study visit: 12.0±11.	From general pediatric clinics in the surrounding community and through newspaper advertisements	6-mercaptopurine, sulfasalazine, mesalamine or asacol metronidazole, corticosteroid	Pediatric -CDAI	BMI was calculated from weight and height	CD:104	233	15.4±4.3	11.9±5.7	19.4±3.2	19.5±4.9	8	Cross-sectional study	America
3. Cravo, M. 2010	Evaluate the presence of metabolic bone disease in patients with CD and to identify potential etiologic factors.	Outpatients with CD, mild to moderate disease	Individuals with a similar age and gender distribution	None of the patients were on steroids at the time of the study and none were hospitalized	Harvey–Bradshaw Index (HBI)	Not mentioned	CD:99 (M:37 F:62)	56 (M:19 F:37)	40±14	42±10	24.5±4.4	24.3±4.1	7	Case–control study	Portugal
4. Geerling, B. J. 2000	Establish a comprehensive picture of the nutritional status in recently diagnosed IBD patients.	Patients diagnosed IBD within 6 months prior to entering the study	Randomly selected from the patient population database of a general health care center	Mesalamine, Azathioprine, Prednisone	CDAI for CD patients, Truelove and Witts' criteria for UC	BMI was calculated from weight and height	IBD:69 CD:23 UC:46	69	CD:30.1±10.2 UC:37.8± 14.7	not mentioned	CD:22.2±2.7 UC:23.1±3.0	CD: 22.2±2.7 UC:24.7±3.5	7	Case–control study	Netherlands
5. Ghoshal, U. C. 2008	Patients with IBD and healthy subjects were evaluated for nutrition using dietary survey, anthropometric and biochemical parameters.	Patients diagnosed IBD	Staff members and healthy relatives of patients	Not mentioned	Truelove-Witt’s for UC, the Harvey Bradshaw activity index for CD	BMI was calculated from weight and height	IBD:62 CD:7 UC:55	41	IBD:35 (16–70)^c^	38 (22–60)^c^	19.8 (13.7–27.5)^c^	23 (17.9–27.2)^c^	8	Case–control study	Northern India
6. Jahnsen, J. 1997	Compare bone mineral density in patients with CD with patients with UC and healthy subjects, and to evaluate possible risk factors for bone loss in IBD.	Patient with IBD.	Age and gender matched normal controls	Corticosteroids, azathioprine, sulfasalazine, 5-ASA	Not mentioned	BMI was calculated from weight and height	CD:60 UC:60	60	CD: 36(21–75) UC: 38(21–75)^c^	36(21–75)^c^	CD: 23.3±4.3 UC: 25.2±5.1	23.4±3.1	8	Cross-sectional study	Norway
7. Mijac, D. D. 2010	Estimate the prevalence of undernutrition and to evaluate methods for routine nutritional assessment of active IBD patients.	Patients with active IBD	Healthy volunteers	Azathioprine, Mesalazine, Prednisone	CDAI for CD, the Mayo score for UC	BMI was calculated from weight and height	IBD:76 UC:53 CD:23	30	IBD: 40.83 ±15.45 UC: 42.31 ± 15.42 CD: 39.21± 15.47	45.10±18.06	IBD:21.35 ±3.65 UC:21.71 ±3.88 CD:20.92 ±2.95	26.55 ±4.76	7	Case–control study	Belgrade
8. Mohamed Hussein, A. A. 2007	Determine the frequency and type of pulmonary dysfunction in patients with UC with respect to disease activity.	Patients with UC	Age and gender matched normal controls living in the same neighborhood	Sulphasalazine,mesalazine,	The Truelove score	Not mentioned	UC:26 (Active UC:20 Remission UC:6)	16	39.5±4	34.7±3	Active UC:17.2±3 Inactive UC:18.4±2.8	23.1±3	8	Prospective study	Egypt
9. Nic Suibhne, T. 2012	Determine the prevalence of overweight and obesity in patients with CD compared with matched healthy controls and to identify disease-specific and generic factors associated with current BMI in this group.	Adult patients with CD for a minimum of 3 months	Age, gender and socioeconomic llymatched healthy controls from non-medical departments and businesses within the hospital's catchment area	Corticosteroid, immosuppressant, 5-ASA, biologics	CDAI	BMI was calculated from weight and height	CD:100	100	35.7 ±10.9	37.9±11.0	25.08±5.5	25.43±3.8	8	Prospective case–control study	Ireland
10. Zoli, G. 1996	Determine energy requirements and the relationship between energy expenditure and growth in adolescents with inactive CD and healthy growing controls.	Patients less than age 20 with onset of disease prior to age 16 and to have been diagnosed for a minimum of two years.	Healthy, growing, age and gender matched adolescents	No subject was currently receiving corticosteroids	CDAI	BMI was calculated from weight and height	CD:10	10	17.6 ±1.4	17.5 ± 1.4	19.2±0.6	23.7±0.6	8	Case–control study	England
11. E. Capristo. 1998	Evaluate the effect of disease localization on the anthropometric and metabolic characteristics of inactive CD.	Patients in clinical remission and not receiving steroid therapy or nutritional support	Age and height matched healthy volunteers	Not receiving steroid therapy	Simplified-CDAI	BMI was calculated from weight and height	CD:43	60	32.0±10.3	33.8±8.7	21.5±1.5	23.7±1.3	7	Case–control study	Italy
12. E. Capristo. 1998.	Evaluate the anthropometric and metabolic characteristics of patients with CD and UC, comparing both groups with healthy volunteers.	Patients in clinical remission not receiving steroid therapy	Age and gender matched healthy volunteers	Not receiving steroid therapy	Simplified-CDAI for CD, Powell-Tuck index for UC	BMI was calculated from weight and height	IBD:34 CD:18 UC:16	20	CD:33.4 (18–60)^c^ UC:40.7 (20–60)^c^	39.4 (18–58)^c^	CD:20.5 (17.2–23.7)^c^ UC:24.3 (20.5–29.7)^c^	23.6 (19.4–26)^c^	7	Cross-sectional study	Italy
13. E. Capristo. 1998.	Measure body composition, whole body glucose uptake and oxidation in CD and UC patients with inactive disease.	Patients in clinical remission not receiving steroid therapy	Age and height matched healthy volunteers	Not receiving steroid therapy	Simplified-CDAI for CD, Powell-Tuck index for UC	BMI was calculated from weight and height	CD:10 UC:10	40	CD: 31.1±7.0 UC:33.4±8.8	36.3±11.2	CD:20.4±1.6 UC:24.0±1.24	23.8±1.85	7	Case–control study	Italy
14. Lucio Cuoco. 2008	Evaluate nutritional status and body mass composition in patients with newly diagnosed CD, and to analyze whether changes in skeletal muscle composition could be attributable to pro-inflammatory cytokines, and to correlate muscle damage with the inflammatory status and intestinal permeability, and circulating bacterial breakdown products of these patients.	Patients with active CD and free of therapy drugs, in particular steroids or immunosuppressive agents	Age and gender matched healthy volunteers	Free of therapy drugs, in particular steroids or immunosuppressive agents	CDAI	BMI was calculated from weight and height	CD:13	20	31(17–49)^c^	not mentioned	19.8±1.2	23.4±1.1	7	Case–control study	Italy
15. Je´roˆme Filippi. 2006	Assess food intake and the status for vitamins and trace elements in nonselected CD patients in clinical remission.	Patients in clinical remission for at least 3 months, under maintenance therapy	Age and gender matched healthy volunteers	5-ASA [mesalamine] and/or azathioprine	CDAI	BMI was calculated from weight and height	CD:54 (M:26 F:28)	25 (M:9 F:16)	39±1.8^b^	37.8±2.7^b^	22.1±0.5^b^	22.1±0.5^b^	7	Case–control study	France
16. Geerling BJ. 1999	Assess body hydration and the distribution of the body water compartments in defined populations of patients with IBD compared with those of matched healthy controls.	Patients with IBD-new and CD-long	Age and gender matched healthy volunteers	Mesalazine, azathioprine, corticosteroids	Truelove-Witt’s index for UC, CDAI for CD	BMI was calculated from weight and height	IBD-new:52 (CD-new:20[M:7,F:13] UC-new:32[M:14,F:18]) CD-long:40[M:17,F:23]	Matched with IBD-new: 52 Matched with CD-long:40	not mentioned	not mentioned	CD-new:22.7±2.5[M:23.1±2.4, F:22.5±2.6] UC-new:22.7±2.4[M: 23.2±2.2,F:22.3±2.6] CD-long:22.8±4.0[M: 22.1±4.1,F:23.2±3.8]	Matched with CD-new: 23.0±2.8[M: 23.2±2.1,F:22.9±3.1] Matched with UC-new:24.7±3.4[M: 26.7±3.6,F:23.2±2.4] Matched with CD-long:24.0±3.3[M:26.2±3.3, F: 22.4±2.2]	8	Case–control study	Netherlands
17. Geerling BJ. 1998	Obtain a comprehensive picture of nutritional status in patients with long-standing CD that was clinically in remission.	Patients with CD for > 10 y in clinical remission and receiving medical treatment during the study	Age and gender matched healthy volunteers	Mesalazine, azathioprine, corticosteroids	CDAI	BMI was calculated from weight and height	CD:32 (M:14 F:18)	32 (M:14 F:18)	CD: 40.0 (34.3–54.0) (M: 49.5 (36.5–56.8) F:39.0 (32.0–48.0)) ^c^	43.8±13.5	CD: 23.2± 3.7 (M: 22.8± 4.1 F: 23.4± 3.5)	24.6±3.6 (M:26.4± 3.5 F:23.3± 3.2)	8	Case–control study	Netherlands
18. Greco Aldo V. 1996	Compare REE and measures of substrate oxidation such as the non- protein respiratory quotient between a homogeneous group of Crohn's patients studied in the same phase of disease activity and a matched control group of healthy volunteers.	Patients in clinical remission receiving a low prednisone dose for a period of at least six months	Age, height and gender matched healthy volunteers	Corticosteroids	CDAI	BMI was calculated from weight and height	CD:20	16	29.50±2.91	30.75±2.15	19.89±0.71	24.77±0.49	7	Case–control study	Italy
19. Geltrude Mingrone.1998	Assess the effect of steroid therapy on body composition, energy expenditure, and fuel selection in CD.	Patients with biopsy-provenileal Crohn disease	Age and height matched healthy volunteers	Prednisone	CDAI	BMI was calculated from weight and height	CD:12 (M:6 F:6) Untreated Crohn disease: (M:3 F:2) Treated Crohn disease (M:3 F:4)	11 (M:6 F:5)	Untreated Crohn disease: 38±14 Treated Crohn disease: 32±14	39±10	Untreated Crohn disease: 17.2±1.9 Treated Crohn disease: 22.8±2.3	24.8±1.21	7	Case–control study	Italy
20. Ste´phane M.Schneider. 2008	Measure the prevalence of sarcopenia in CD patients in remission and uncover its relationship with osteopenia.	Patients with CD in clinical remission	Age and height matched healthy volunteers	Corticosteroids, oral mesalamine, azathioprine, TNF-alpha antagonists (infliximab)	CDAI	BMI was calculated from weight and height	CD:82	50	36.2±13.9	39.2±13.3	21.1±3.4	22.2±2.5	7	Case–control study	France
21. Jean-Baptiste Wiroth. 2005	Assess muscle strength and endurance in CD patients in clinical remission and the influencing factors.	CD outpatients, in clinical remission not receiving GCs for at least 2 months	Age and gender matched healthy volunteers	No patient had been receiving GCs for at least 2 months	CDAI	BMI was calculated from weight and height	CD:41 (M:17 F:24)	25 (M:10 F:15)	CD:37.4±9.5 (M:38±11.8 F:37.4±9.5)	37.0 (13.0 (M:43.6±13.1 F:32.6±11.2)	CD:22.1±3.6 (M:22.1±3.5 F:22.1±3.7)	22.5±2.3 (M:24.0±2.4 F:21.4±1.6)	7	Case–control study	France
22. Sakellariou, G. T. 2006.	Determine the degree of decreased bone density in steroid naïve young male patients with inflammatory bowel disease and to unmask possible risk factors.	Young male patients aged over 20 years with recently diagnosed IBD	Age and gender matched healthy volunteers	Corticosteroid	Not mentioned	BMI was calculated from weight and height	IBD:32 (CD:18 UC:14)	20	IBD:26±4.8 (CD:26.3±5 UC:25.8±4.6)	24.6±6.2	IBD:24.1±4.3 (CD:24.1±4.6 UC:24.2±4.2)	23.2±4.5	7	Case–control study	Greece
23. Sally L James. 2014	Determine how dietary non-starch polysaccharide (NSP) and resistant starch (RS) is used in patients with UC and assess the tolerability of such a dietary change.	UC patients in remission over the age of 18 years	Healthy controls over the age of 18 years	Oral aminosalicylates, oral corticosteroids or thiopurines	Colitis activity index (CAI) ≤4	BMI was calculated from weight and height	UC:19	10	38 (18–72)	41 (26–66)	25.8±1.1	22.4± 0.7	8	Randomised, cross-over single-blinded controlled study	Australian New Zealand
24. Mahmoud Sajjadi. 2015	Investigate the association of serum adenosine deaminase activity and disease activity in Crohn’s disease patients.	CD patients	Age and gender matched healthy volunteers	Not mentioned	CDAI	BMI was calculated from weight and height	Active CD:15 Remission CD:15	15	Active 39.4±14.4 Remission 34.2±10.4	33.7±5.7	Active 20.9±4.1 Remission 25.3±5.3	25.3±5.2	8	Cross-sectional study	Iran

^a^Mean ± SD

^b^Mean ± SEM

^c^Median and range, IBD: inflammatory bowel diseases, CD: crohn’s disease, UC: ulcerative colitis, M: male, F: female.

### Primary Meta-analysis

**Fig 2:**
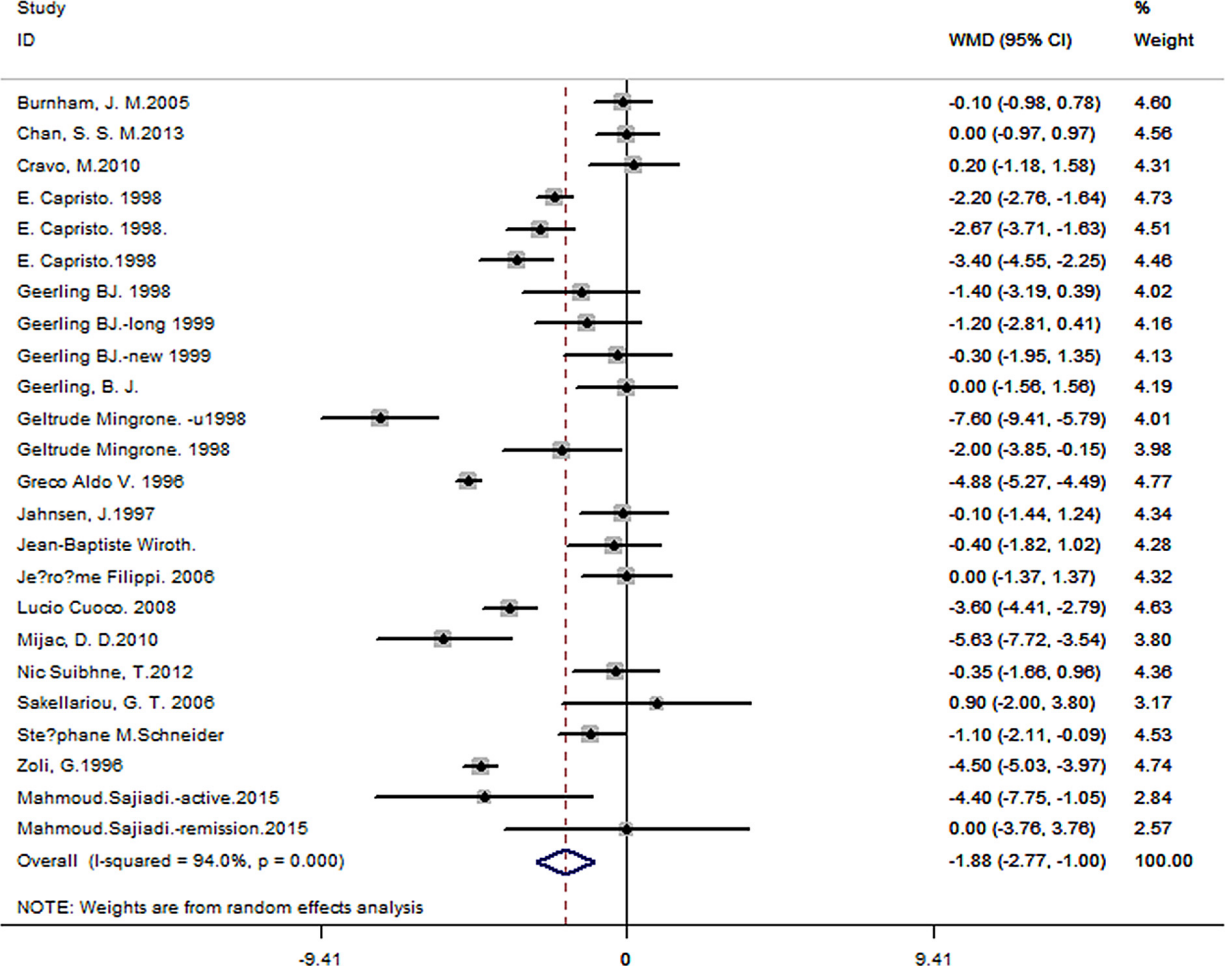
Forest plot of the association between BMI and CD.

**Fig 3:**
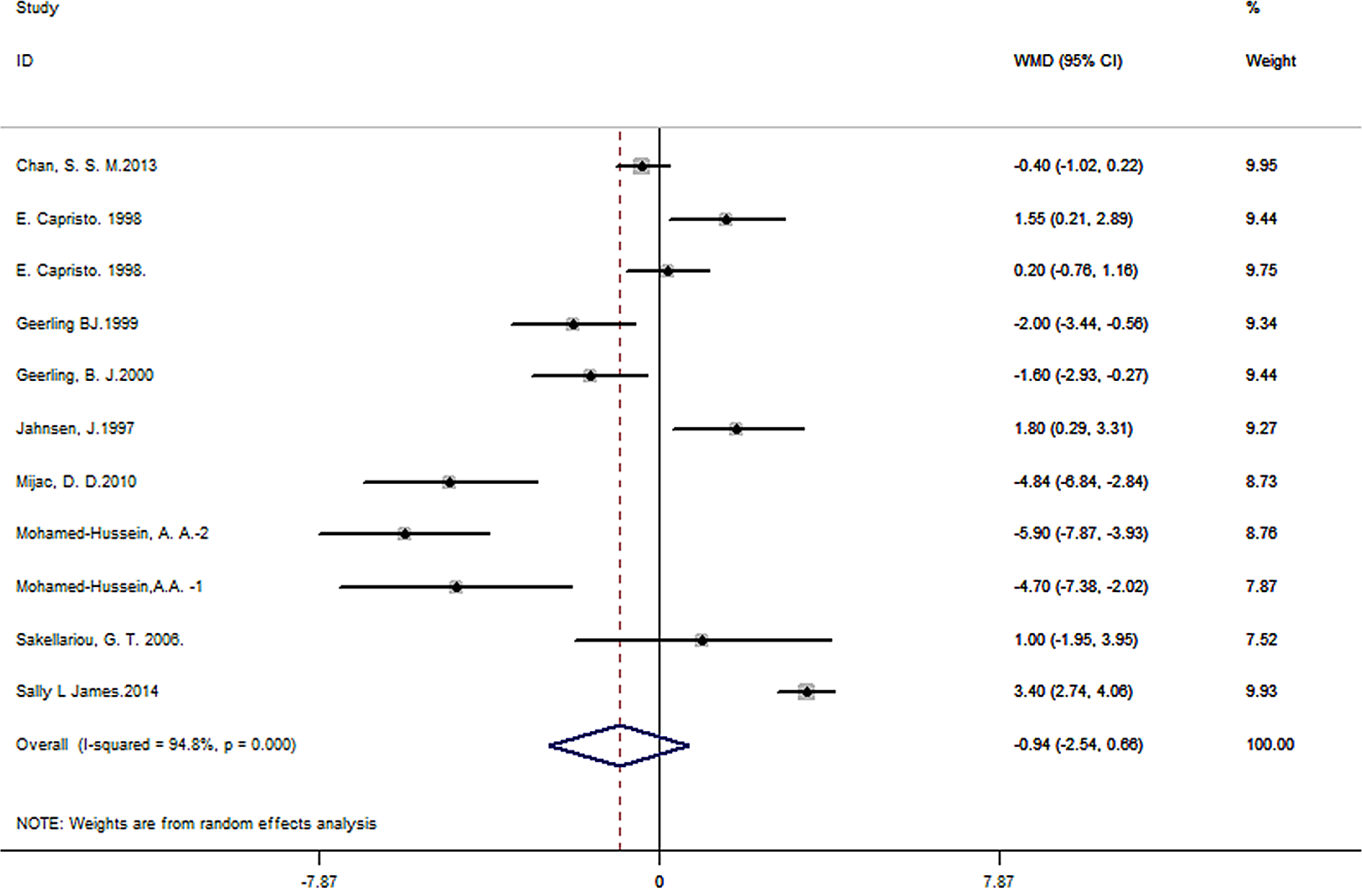
Forest plot of the association between BMI and UC.

**Fig 4:**
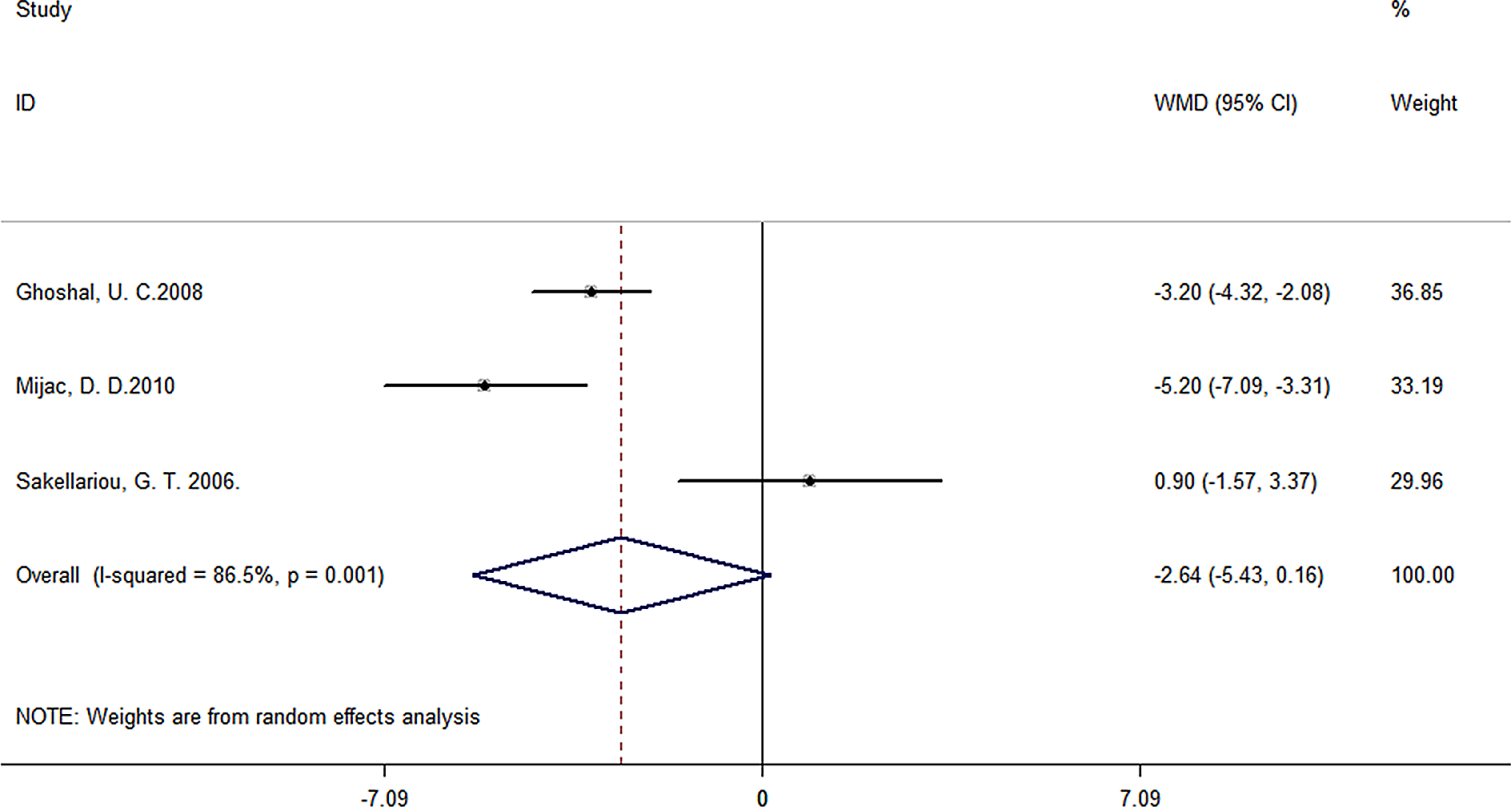
Forest plot of the association between BMI and IBD.

A total of 24 studies containing 1442 patients and 2059 controls were included. Pooled data showed significant BMI difference of -1.88 for CD patients compared to controls (95% CI -2.77 to -1.00, *P*< 0.001) (Fig 2), while no obvious difference was observed for -0.94 in UC patients (95% CI -2.54 to 0.66, *P*< 0.001) (Fig 3) or undefined IBD patients (WMD of -2.64, 95% CI -5.43 to 0.16, *P* = 0.064) (Fig 4). Great heterogeneity was found in primary meta-analysis (*P*< 0.001 for CD, UC as well as non-identified group). There was no evidence of publication bias in primary meta-analysis (*P* value of Begg’s test: 0.735 for CD, 0.276 for UC and 1.000 for non-identified group). Sensitivity analyses indicated that pooled estimate was not excessively changed by any individual studies (Tables 2–4, S1 Fig, S2 Fig).

**Table 2:** Sensitivity analysis of included studies about CD.

Study omitted	Estimate	95% Confident Interval	
Burnham, J. M.2005	-1.972	-2.855	-1.088
Chan, S. S. M.2013	-1.976	-2.860	-1.091
Cravo, M.2010	-1.979	-2.871	-1.087
E. Capristo. 1998	-1.867	-2.829	-0.905
E. Capristo. 1998	-1.847	-2.769	-0.924
E. Capristo. 1998	-1.813	-2.732	-0.894
Geerling BJ. 1998	-1.905	-2.810	-0.999
Geerling BJ.-long	-1.914	-2.820	-1.008
Geerling BJ.-new	-1.953	-2.852	-1.053
Geerling, B. J.	-1.967	-2.863	-1.071
Geltrude Mingrone.–u1998	-1.648	-2.526	-0.769
Geltrude Mingrone. 1998	-1.879	-2.786	-0.972
Greco Aldo V. 1996	-1.734	-2.568	-0.901
Jahnsen, J.1997	-1.966	-2.861	-1.070
Jean-Baptiste Wiroth.	-1.951	-2.851	-1.051
Je^ro^me Filippi. 2006	-1.970	-2.864	-1.075
Lucio Cuoco. 2008	-1.800	-2.733	-0.868
Mijac, D. D.2010	-1.737	-2.635	-0.838
Nic Suibhne, T.2012	-1.954	-2.853	-1.056
Sakellariou, G. T. 2006	-1.976	-2.869	-1.082
Ste^phane M.Schneider	-1.921	-2.831	-1.011
Zoli, G.1996	-1.754	-2.679	-0.830
Mahmoud.Sajiadi.-active.2015	-1.811	-2.708	-0.913
Mahmoud.Sajiadi.-remission.2015	-1.934	-2.829	-1.040
Combined	-1.884	-2.767	-1.002

CD: crohn’s disease.

**Table 3:** Sensitivity analysis of included studies about UC.

Study omitted	Estimate	95% Confident Interval	
Chan, S. S. M.2013	-1.030	-2.983	0.924
E. Capristo. 1998	-1.209	-2.954	0.537
E. Capristo. 1998	-1.081	-2.912	0.749
Geerling BJ.1999	-0.835	-2.533	0.863
Geerling, B. J.2000	-0.878	-2.596	0.841
Jahnsen, J.1997	-1.227	-2.954	0.499
Mijac, D. D.2010	-0.558	-2.150	1.033
Mohamed-Hussein, A. A.-2	-0.447	-1.977	1.083
Mohamed-Hussein,A.A	-0.617	-2.246	1.012
Sakellariou, G. T. 2006	-1.102	-2.785	0.581
Sally L James.2014	-1.376	-2.704	-0.047
Combined	-0.941	-2.543	0.660

UC: ulcerative colitis.

**Table 4:** Sensitivity analysis of included studies about IBD.

Study omitted	Estimate	95% Confident Interval	
Ghoshal, U. C.2008	-2.204	-8.181	3.773
Mijac, D. D.2010	-1.304	-5.311	2.702
Sakellariou, G. T. 2006	-4.048	-5.986	-2.111
Combined	-2.636	-5.426	0.155

IBD: inflammatory bowel diseases.

### Subgroup analysis

#### Medical therapy

**Fig 5:**
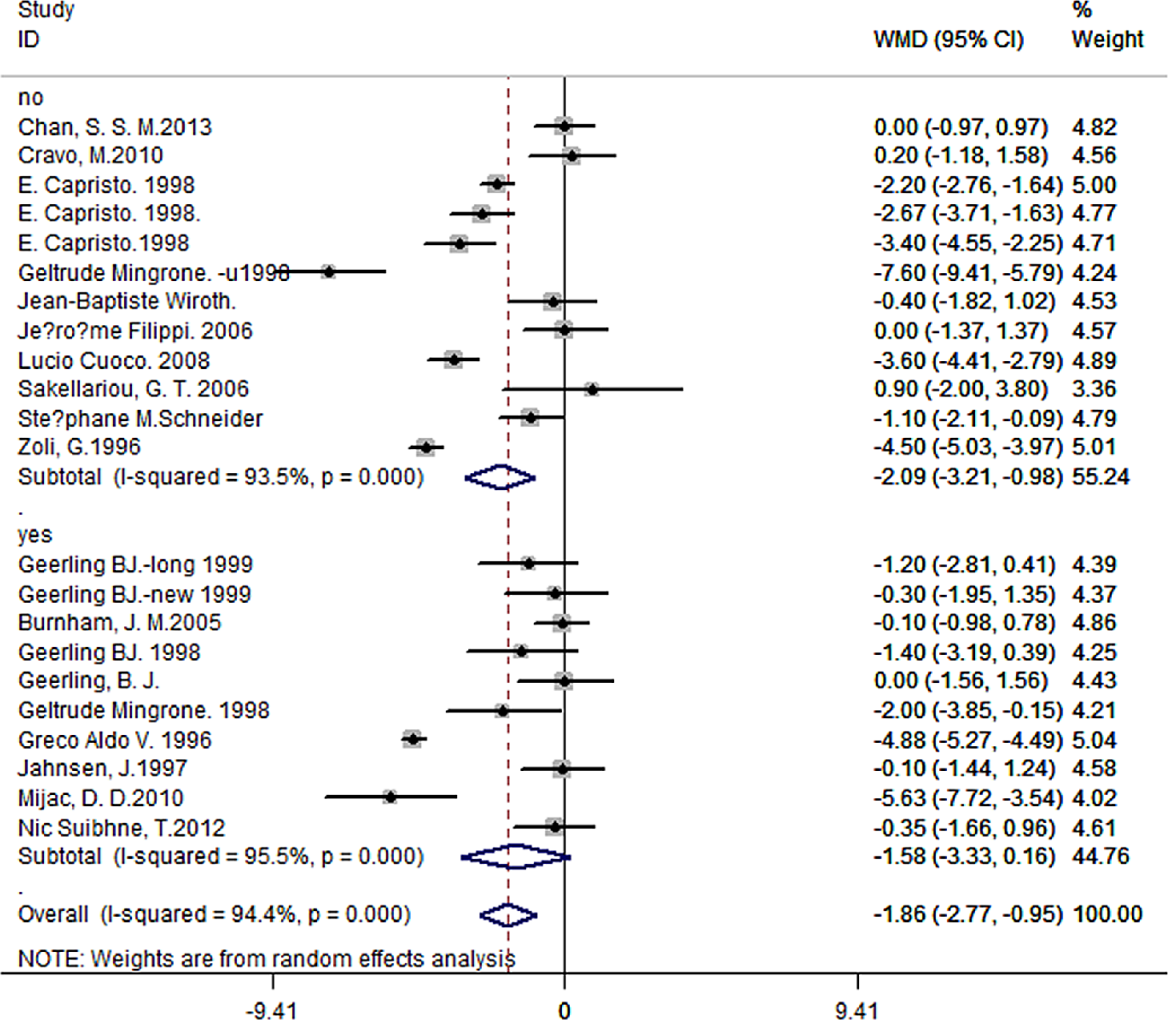
Forest plot of subgroup analysis of BMI in CD patients with or without medicine therapy.

Among the 21 articles enrolled CD patients, 9 articles containing 429 patients and 565 controls mentioned the current use of therapy, like corticosteroid, azathioprine, mesalamine, or TNF-alpha antagonist. 468 patients and 637 controls in 12 studies hadn’t taken any medicine. Pooled data showed significantly different BMI in patients without therapy compared to controls (WMD = -2.09, 95% CI -3.21 to -0.98, *P* < 0.001), difference of -1.58 in BMI in patients with therapy (95% CI -3.33 to 0.16, P < 0.001) (Fig 5). There was no significant difference between the two subgroups (*P* = 0.63).

**Fig 6:**
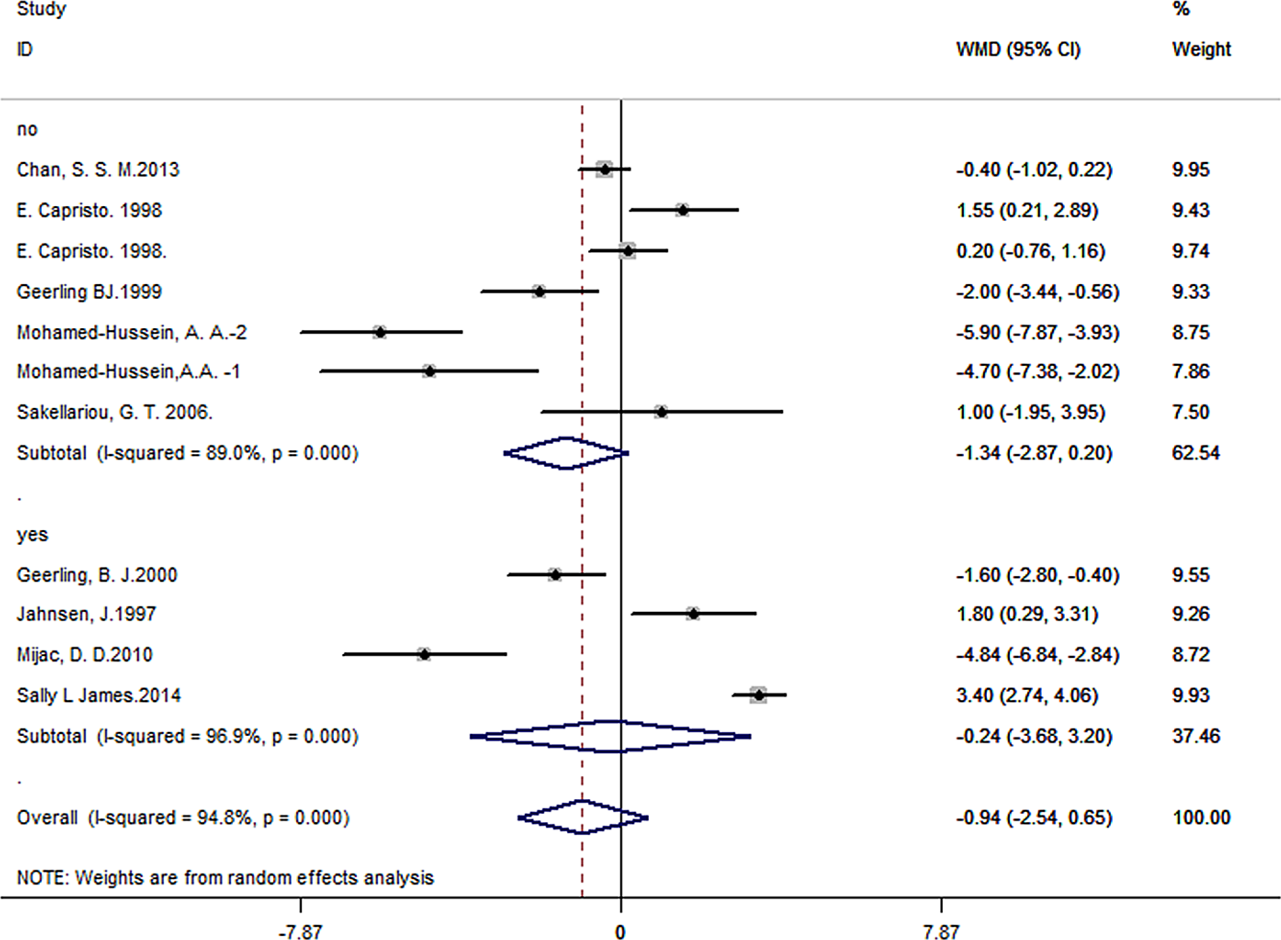
Forest plot of subgroup analysis of BMI in UC patients with or without medicine therapy.

There were 10 studies concerning UC patients. 178 patients from four studies had received therapy, like corticosteroid, azathioprine or mesalamine, during the studies and a total of 169 people were included in the control groups. 275 patients and 820 controls in six studies hadn’t taken any medicine. We observed no significant difference of BMI in patients without medical therapy (pooled WMD = -1.34, 95% CI -2.87 to 0.20, *P* = 0.089), and in BMI in patients with medical therapy (pooled WMD = -0.24, 95% CI -3.68 to 3.20, *P* = 0.891), compared with controls. There was no significant difference between the two subgroups (*P* = 0.57) (Fig 6).

#### Disease state

**Fig 7:**
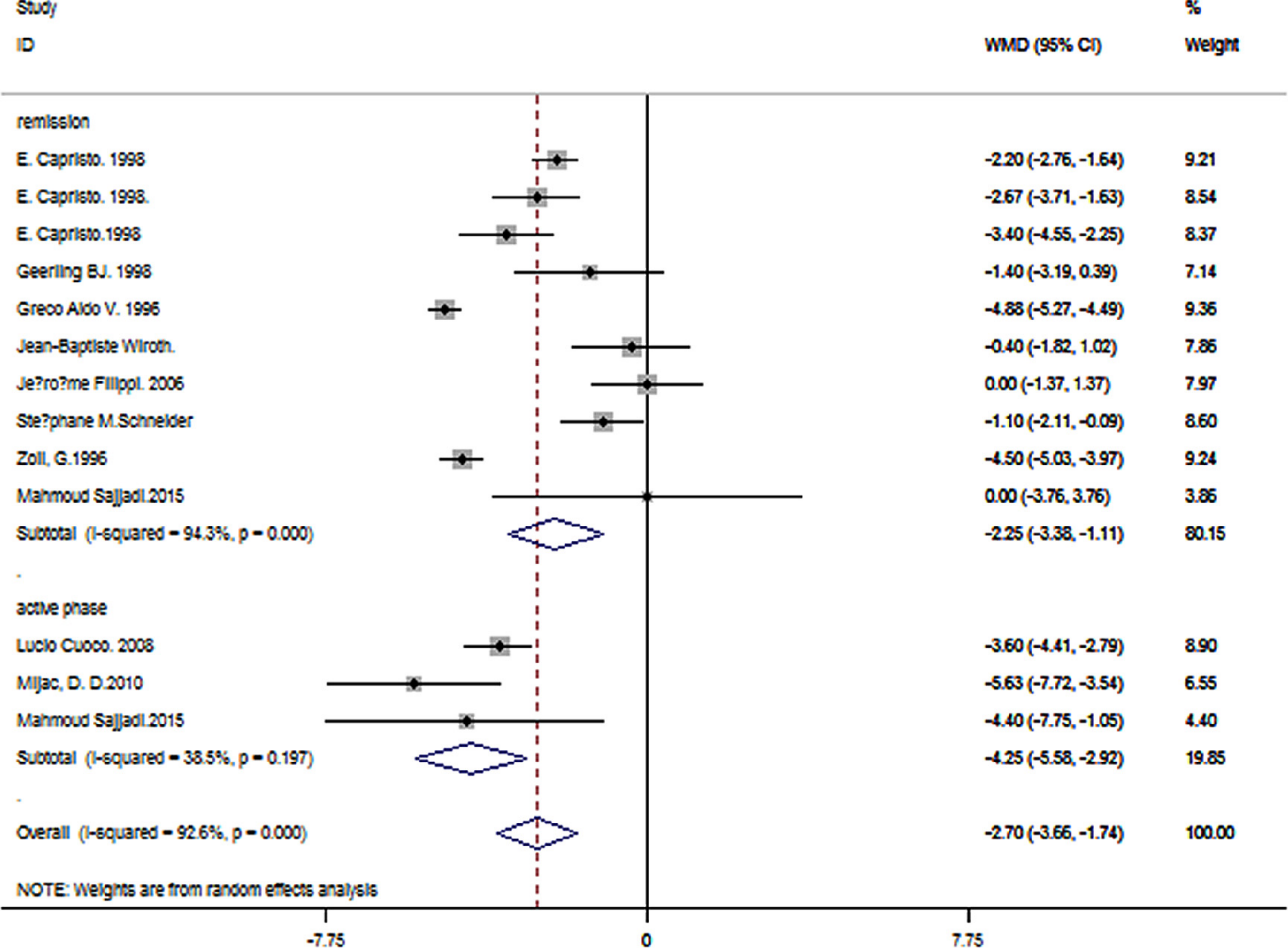
Forest plot of subgroup analysis of BMI in CD patients in active or remission phase.

Crohn’s Disease Activity Index (CDAI), Simplified Crohn’s Disease Activity Index, Pediatric Crohn’s Disease Activity Index (PCDAI) or Harvey–Bradshaw Index (HBI) were used in different studies to assess CD patients’ disease activity. Active phase was defined as CDAI score over 150, below was categorized as in remission. 344 patients in remission and 303 controls were enrolled in 11 studies. A significant difference of BMI was suggested (pooled WMD = -2.25, 95% CI -3.38 to -1.11, P < 0.001). Three studies focused on 51 CD patients in active phase and 65 healthy controls. Pooled data showed a significant different BMI in patients in active phase (pooled WMD = -4.25, 95% CI -5.58 to -2.92, *P* < 0.001). There was a significant difference between the two subgroups (P = 0.03) (Fig 7).

**Fig 8:**
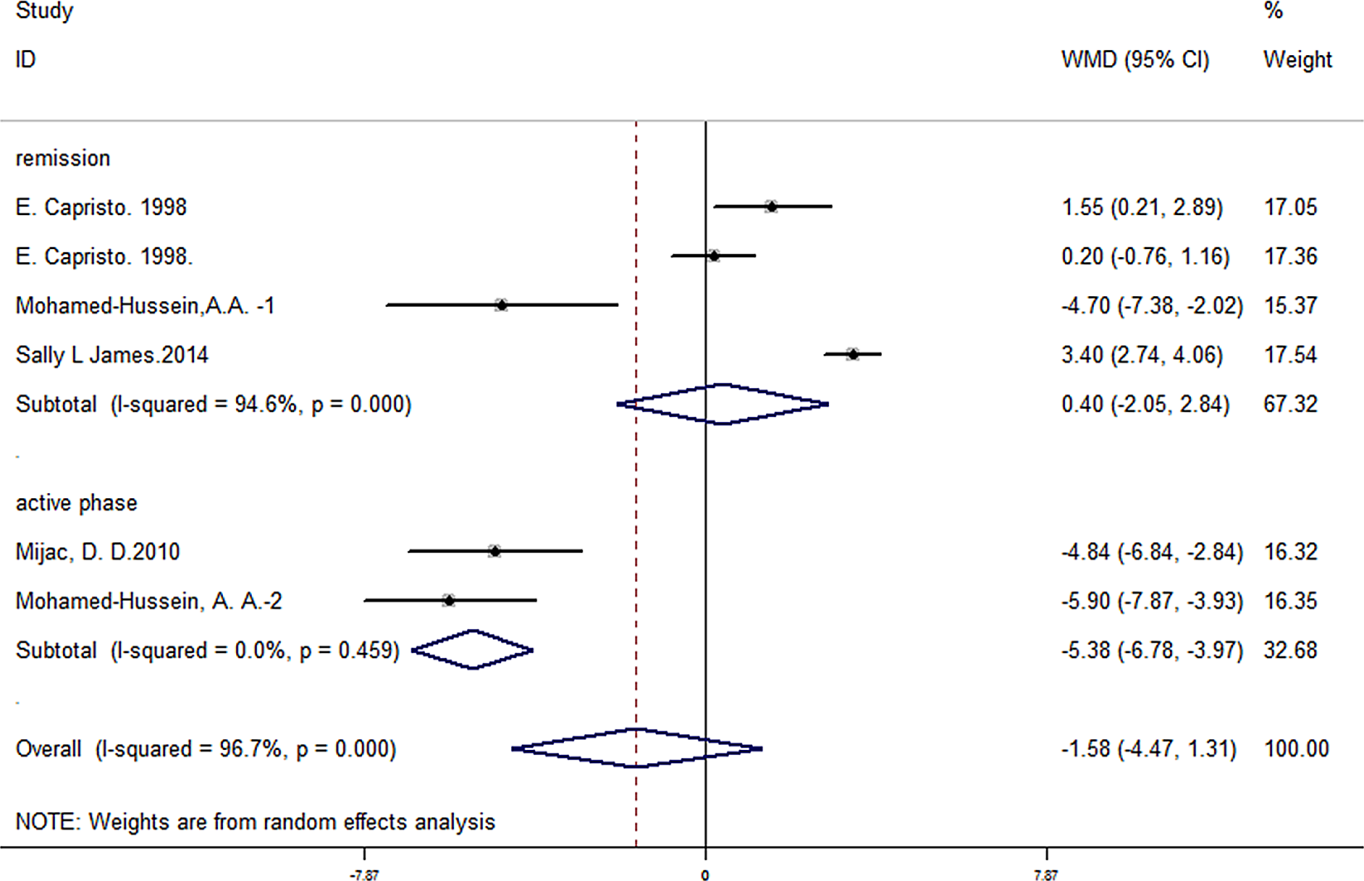
Forest plot of subgroup analysis of BMI in UC patients in active or remission phase.

Truelove-Witts’ index, the Mayo score, Powell-Tuck index or Colitis activity index were used in different studies to assess UC patients’ disease activity. Two articles contained 73 UC patients in active phase and 565 controls. A significant difference of BMI was found in patients in active phase (pooled WMD = -5.38, 95% CI -6.78 to -3.97, P < 0.001). Pooled data showed no significant difference of 0.40 in BMI in 51 patients in remission and 86 controls (95% CI -2.05 to 2.84, P = 0.752) from four studies. There was a significant difference between two subgroups (P = 0.001) (Fig 8).

## Discussion

The present meta-analysis is the first to assess the association between BMI and IBD patients. The inclusive finding of our systematic review is that both UC and CD patients had consequential lower BMI than controls. This might be illustrated by reduced dietary intake, malabsorption. Metabolic disturbances, such as increased energy expenditure, decreased respiratory quotient, and increased substrate oxidation rates, may also be a reason causing patients’ loss of glucose, protein and fat [40, 42]. In prior works [15, 33], researchers have proven that BMI was one of the most predictive parameters to assess the nutritional status in IBD patients. Meanwhile BMI was quite simple and convenient to acquire. Since malnutrition is frequently found in patients with IBD, physicians should be more aware of this concern in patients with low BMI. Consequently, IBD patients are most likely at an increased risk to develop osteopenia and osteoporosis. More studies are needed to elucidate whether nutritional supplementation in IBD patients may improve the clinical course of the disease or not [21].

To some degree, medical therapy, including corticosteroid, azathioprine, mesalamine and TNF-alpha antagonists, could improve CD patients’ BMI. Without medicine, patients’ BMI was significantly lower than non-IBD controls’ while with medical management, the difference was not significant. Medical therapy could help improve patients’ health condition by relieving nutrition loss or delay disease progression [19]. In a preceding study, decreased bone mass was already present at the time of diagnosis inpatients with CD. There was no significant deference of BMI in pooled data among the subgroups of CD patients with and without therapy versus controls shown in Geltrude Mingrone’s study [24], BMI of treated Crohn disease patients is significantly higher when compared with untreated Crohn disease patients (*P* = 0.004). In a recent study of postmenopausal women in the USA, a significant linear trend across BMI categories with bone mineral density was observed [44]. In a meta-analysis of 60 000 patients from 11 prospective studies, relative risk of fracture rose from 1.4 in females with BMI of 20 to 2.2 in females with BMI of 15 [45]. Patients’ diet also affects on bone metabolism since calcium, vitamin D, and vitamin K is necessary for bone metabolism [46]. Chronic inflammation, prolonged use of steroids, as well as low intake of calcium and vitamin D is other most frequently implicated factors [47].

However, we did not observe similar result in UC patients who might be due to the relative limited number of studies included.

CD patients suffered more from subordinate BMI both in active phase and in remission than controls. UC patients had a lower BMI in active phase, while in remission, there was no difference compared with controls. It indicated that BMI could be a marker of disease state which could further develop into a prognosis predictor and treatment efficacy parameter. Discrepant conclusions were achieved from previous studies. Denia Stabroth-Akil’ team found that high BMI had a positive effect on the prognosis, whereas low BMI pointed to a more severe course of the disease after a retrospective analysis of data from 202 UC patients [48]. However, the outcome from Millie D. Long’s research was quite opposite. Obese IBD patients may have a more severe disease course and increased need for surgery [49]. In a retrospective cohort of 124 IBD patients treating with infliximab, researchers performed a multivariable logistic regression between BMI category and response to infliximab. Obesity was associated with an earlier time to loss of response to infliximab [50]. In Avegail Flores’ study, IBD patients with low BMI were more likely to receive anti-TNF treatment, undergo surgery, or experience a hospitalization than patients with high BMI [51]. Continued observation of BMI might help appraise medicine efficacy and contribute instruction in medical therapy readjustment.

Obesity has been discovered to be associated with excess adipocyte hypertrophy generating a proinflammatory state through secretion of inflammatory cytokines and chemokines, including interleukin (IL)-1β, IL-6, IL-8, monocyte chemoattractant factor,[52] tumor necrosis factor-α [53], and C-reactive protein [54]. These bio-factors might be closely related to pathogenesis of IBD. In the existing analysis, there is one prospective study observing IBD incidence among patients with various BMI. After following 300,724 people for an average of 4.5 years (range from 1.6 to 15.6 years), researchers have revealed that BMI is not associated with IBD morbidity, neither CD nor UC [18], which might be explained by that the results of existent study may be modified by certain factors, such as population, follow-up time, age and geographic distribution of study population, so conclusions of whether BMI contributes to IBD development can yet be drawn until more researches have been done.

In addition to being a potential prognosis marker or risk factor for IBD, BMI may also affect severity of complication in IBD. Increasing data are emerging both in patients with IBD and in the healthy population designating that low BMI is an indicative independent risk factor for osteoporosis.

Our study has a couple of strengths. First of all, this is the first systematic review and meta-analysis with regard to BMI in IBD patients. Second, most of the incorporated studies were of high methodological quality. Third, no publication bias was ascertained, and subgroup analysis was applied to assess the role of therapy and disease stage.

However, inevitable limitations existed in our analysis. Proportionately large heterogeneity may influence the combined result and thus the conclusion. Besides, small sample size may affect the outcome of the analysis. In addition, not enough studies were included to conduct quantity’s analysis in certain concerns. Furthermore, investigation is needed to figure out the complicated role of BMI in IBD patients.

In conclusion, our meta-analysis revealed that IBD patients had lower BMI than normal controls, and this dissimilarity might be rationalized by disease remission and medical therapy, indicating that BMI may serve as an easily accessible factor in IBD prognosis and treatment effectiveness.

## Supporting Information

S1 Fig Sensitivity analysis of included studies about CD. (TIF)

S2 Fig Sensitivity analysis of included studies about UC. (TIF)
