# Supplementary figures and images for: Body Mass Index Is Associated with Inflammatory Bowel Disease: A Systematic Review and Meta-Analysis
Source: PLoS One. 2015 Dec 14;10(12):e0144872. doi: 10.1371/journal.pone.0144872 (PMC4684381; doi:10.1371/journal.pone.0144872)

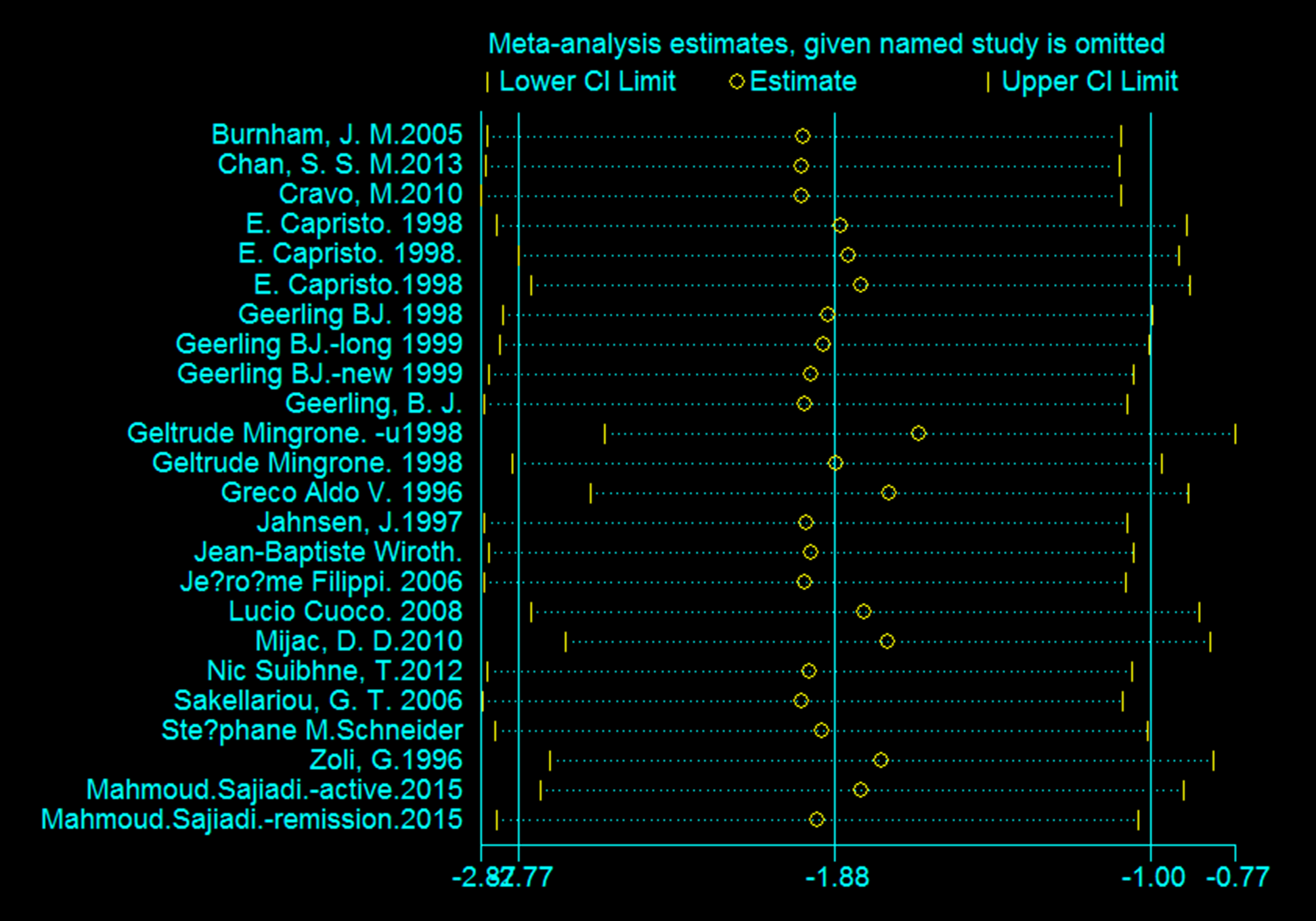

Supplement: S1 Fig — (TIF) [file pone.0144872.s001.tif]

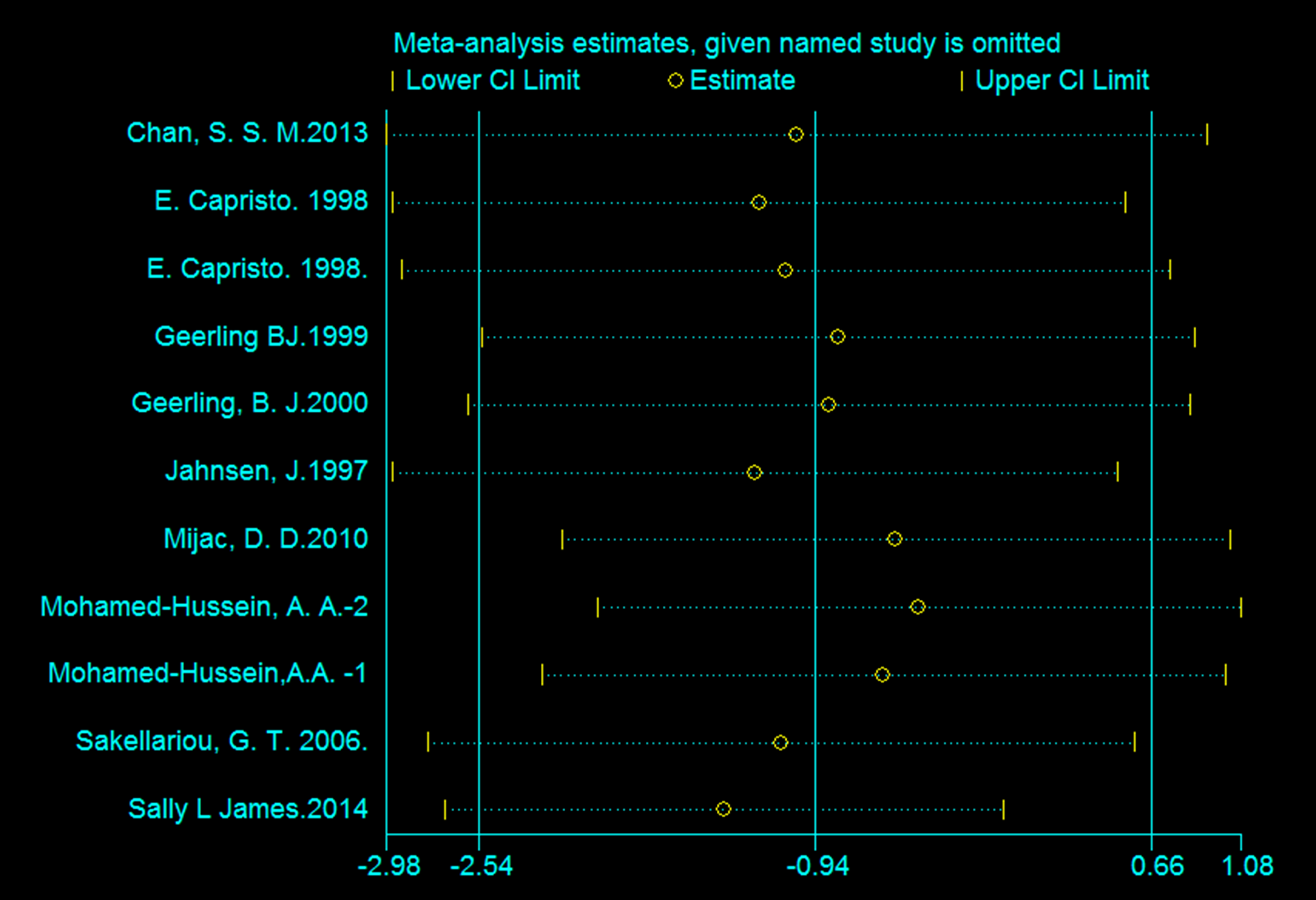

Supplement: S2 Fig — (TIF) [file pone.0144872.s002.tif]
